# Degradation of the Intermediate Band Caused by Disorder in Quantum-Dot Intermediate-Band Solar Cells

**DOI:** 10.3390/mi17091005

**Published:** 2026-08-25

**Authors:** Lucas Cuadra, Jorge Pérez-Aracil, Sancho Salcedo-Sanz

**Affiliations:** Department of Signal Processing and Communications, University of Alcalá, 28805 Madrid, Spain; jorge.perezaracil@uah.es (J.P.-A.); sancho.salcedo@uah.es (S.S.-S.)

**Keywords:** colloidal quantum dots, intermediate-band solar cells, structural disorder, participation ratio, tight-binding model, localization

## Abstract

Intermediate band solar cells require in-gap electronic states that remain sufficiently extended to sustain collective electronic coupling and facilitate carrier motion. We investigate how structural disorder affects an intermediate band formed by coupled colloidal quantum dots embedded in a perovskite-like matrix. The system is represented by a single-orbital tight-binding Hamiltonian on a dilated face-centered-cubic lattice containing 4000 quantum dots, with system sizes between 1372 and 5324 in the finite-size analysis. Radius dispersion modifies on-site energies and hopping amplitudes, whereas positional disorder acts mainly through variations in interdot separation. Eigenstate extension is quantified using the normalized participation ratio. To distinguish spectral broadening from the loss of useful extended states, we also evaluate the mean participation of a contiguous threshold-defined spectral core, its relative energy width, and their product as a combined robustness descriptor. For the adopted baseline parameter set, degradation is energy selective: states near the spectral edges lose participation before states near the band center. At equal nominal amplitudes, radius disorder produces a stronger response than positional disorder, although the two amplitudes do not represent equal realized variances. A positional-disorder amplitude of 0.05 retains approximately 86% of the ordered-reference value of the combined descriptor, whereas a radius-disorder amplitude of 0.05 reduces it to about 22%; when both amplitudes are 0.05, about 14% remains. The model displays a comparatively robust regime near $\sigma_{R}=0.02$, a model-dependent crossover around $\sigma_{R}=0.03$–0.04, and strong degradation at larger values. Finite-size results are consistent with near-extensive scaling of the effective core participation number over the simulated sizes, but do not establish a thermodynamic mobility edge. These findings identify quantum-dot size uniformity as the more restrictive model control variable for preserving an extended intermediate-band core.

## 1. Introduction

**Figure 1:**
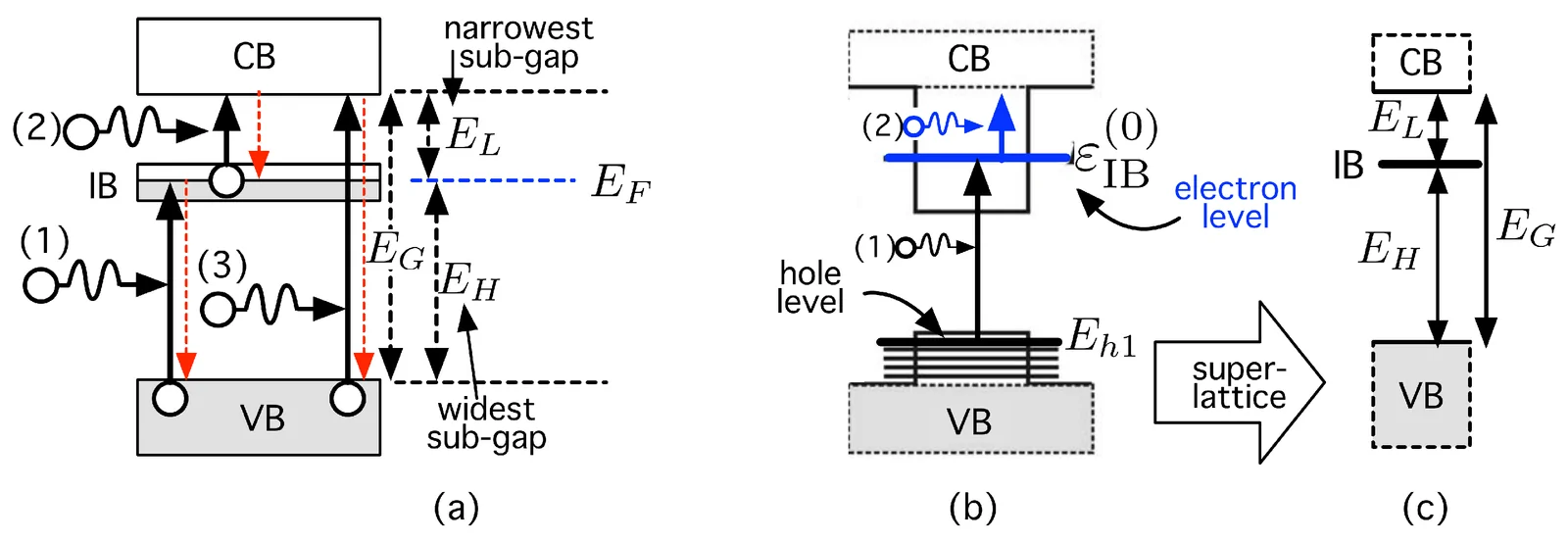
(**a**) Simplified illustration of the gaps and operation of an IBSC. The absorption of a sub-bandgap photon (1) pumps an electron from the valence band (VB) to the IB while that of photon (2) excites another electron from the IB to the conduction band (CB) [1]. This extra, two-step, two-photon photocurrent is added to the conventional one generated by photons like (3) with $E>E_{G}$. (**b**) Single QD. (**c**) Resulting IB material. The IB should ideally arise from the discrete electron level $\varepsilon_{\mathrm{IB}}^{\left(0\right)}$ in a superlattice of QDs. See the main text for further details.

The intermediate band solar cell (IBSC), originally proposed by Luque and Martí [1], was conceived as a route to overcome one of the fundamental limitations of conventional single-gap solar cells [2,3,4]. In a semiconductor with bandgap $E_{G}$, photons with energies below the gap (“sub-bandgap photons”), $E<E_{G}$, cannot promote electrons directly from the valence band (VB) to the conduction band (CB) and therefore do not contribute to conventional electron–hole pair generation. In an IBSC, an isolated intermediate band (IB) is introduced inside the host bandgap, thereby enabling the absorption of sub-bandgap photons through two sequential optical transitions, as illustrated in Figure 1a. A first photon promotes an electron from the VB to the IB, while a second photon excites an electron from the IB to the CB. The resulting two-photon photocurrent supplements the conventional photocurrent generated by photons with $E>E_{G}$ [1]. A defining feature of the ideal IBSC concept is that this additional photocurrent can be generated without reducing the output voltage. The voltage remains limited by the total bandgap $E_{G}$, rather than by either of the two sub-bandgaps, $E_{L}$ and $E_{H}$, created by the IB. Two-photon photocurrent (TPPC) and voltage preservation (VP) therefore constitute the two fundamental operating principles of the IBSC. Both mechanisms have been experimentally investigated [5,6,7,8,9,10,11,12,13] in devices based on stacks of epitaxial quantum dot (QD) layers, including demonstrations at room temperature [10]. Its limiting efficiency—using detailed balance concepts in the radiative limit according to the Second Law of Thermodynamics [14,15]—has been found to be 63.2% (under fully concentrated sunlight) [1], much higher than that of the conventional cell (40.7% [16]).

QDs provide a natural platform for implementing the IB concept because three-dimensional quantum confinement produces discrete, atom-like energy levels (Figure 1b) separated by zero density of states (DOS) [17,18,19]. As shown in Figure 1c, the IB should ideally arise from the discrete electron level $\varepsilon_{\mathrm{IB}}^{\left(0\right)}$ in a superlattice of QDs [20,21].

Epitaxially grown QD-IBSCs have provided important experimental evidence for the operating principles of the IBSC. Nevertheless, they face several limitations. The energy separation between confined QD states and the conduction band may be insufficient to suppress thermal escape at room temperature, which can compromise voltage preservation [22,23]. In addition, the optical transitions involving the IB are generally much weaker than the direct VB–CB transition [24,25,26]. One contributing factor is the relatively low QD density achievable in conventional epitaxial structures, typically of the order of $10^{15}$–$10^{16}\;{\mathrm{cm}}^{-3}$, which limits the absorption associated with the intermediate states.

Colloidal quantum dots (CQDs) offer an alternative route to overcome some of these constraints. First, the phonon-bottleneck effect detected in doped CQDs [27,28] may suppress or slow intraband carrier relaxation. Second, CQDs can be densely packed (∼$10^{19}$–$10^{20}$ cm^−3^) and heavily n-doped, leading to strong absorption coefficients (∼$10^{4}$ cm^−1^). In particular, they can be synthesized by scalable solution-based methods [29] with accurate control over QD size, composition, and surface chemistry [30,31]. Moreover, ligand engineering [32,33,34] enables the passivation of surface defects [35,36,37,38,39] and the modification of interdot separation (by ligand exchange [40,41,42]), two factors that directly influence electronic coupling and carrier lifetime in solar cells [34]. These beneficial properties have made CQDs attractive building blocks [43,44] for photovoltaic and other optoelectronic applications [17,45,46,47]. Readers interested in an updated and illustrative review of the application of QDs to IBSCs are referred to [48].

Of particular interest is the architecture commonly referred to as colloidal quantum dots in matrix (CQDM), or quantum-dot-in-perovskite (QDiP) solids. In the seminal work by Ning et al. [49], PbS CQDs were embedded into a MAPbI_3_ perovskite matrix through a solution-processed heteroepitaxial approach. In this architecture, the host matrix provides the valence- and conduction-band states and may support comparatively efficient carrier transport [26], whereas the confined electronic states of the embedded CQDs produce states within the bandgap. PbS CQDs are particularly attractive in this context because their energy levels are strongly tunable through dot size [30,31]. The CQDM architecture therefore combines the high density and spectral tunability of CQDs with transport pathways provided by a crystalline semiconductor matrix [50,51,52,53,54,55].

A substantial body of experimental work has subsequently established the potential of this material platform. CQD–perovskite systems have been investigated in photovoltaic devices, infrared light-emitting diodes, and photodetectors [56,57,58]. Most importantly in the present context, Hosokawa et al. [54] demonstrated the sequential two-step photoresponse at room temperature using PbS CQDs densely dispersed in a CH_3_NH_3_PbBr_3_ matrix. Lattice anchoring between lead-chalcogenide CQDs and perovskites has also been shown to suppress aggregation, improve structural stability, and enhance carrier mobility relative to pure CQD films [59]. More recently, the influence of PbS CQD size and concentration on sub-bandgap sensitization and the possible development of an intermediate miniband has been explored in MAPbI_3_ matrices [60], while thermal isolation of QD-derived intermediate states from the host conduction band has been demonstrated through bandgap engineering [61]. These results establish the CQDM approach as a physically relevant platform to investigate and put into practice the concept of IB devices.

From the theoretical side, Alexandre et al. [62] developed a single-band effective-mass framework for describing the confined electron states and the optical absorption of CQDs embedded in a wider-bandgap semiconductor host. Their model has shown that QD size is the dominant parameter controlling the number and energy spacing of confined states, whereas variations in the QD–host potential barrier mainly produce an approximately rigid shift of their energies. This article also shows that, at sufficiently high QD densities, quantum-confinement-mediated absorption coefficients may approach those characteristic of bulk photovoltaic semiconductors. Importantly, that framework addresses the design of the confined spectrum and its associated optical response. Size dispersion enters principally through the broadening of the resulting absorption features.

The present work addresses a different and complementary question. In a real CQDM material, the existence of confined states at suitable energies does not by itself guarantee the formation of an electronically useful IB. The individual QD states must couple strongly enough to form collective eigenstates with sufficient spatial extension. Structural inhomogeneity may disrupt this coupling and progressively transform an extended miniband into weakly connected or localized states. In this case, the density of states or the total spectral width may still suggest the presence of an intermediate band even though a substantial fraction of its states no longer extends over the CQD ensemble. This distinction is particularly relevant for carrier motion and motivates an explicit analysis of the spatial character of the IB eigenstates.

A high density of QDs does not, by itself, guarantee the formation of an intermediate band in the physically relevant sense of a set of mutually coupled electronic states with sufficient spatial extension. A dense ensemble may still give rise to a collection of weakly coupled or strongly localized in-gap states rather than to a genuinely collective miniband. This distinction is particularly important from a photovoltaic perspective, because localized states within the semiconductor bandgap can acquire a defect-like character and provide efficient non-radiative recombination pathways.

This issue is closely related to the well-known detrimental role of deep mid-gap impurity levels. Deep levels are typically associated with strongly localized electronic wavefunctions generated by short-range impurity potentials [63]. Their strong spatial localization enhances the local electron–lattice interaction and can facilitate non-radiative carrier capture through multiphonon emission [63]. Such deep centers may therefore act as efficient recombination centers, reducing minority-carrier lifetimes and ultimately degrading photovoltaic performance.

This analogy should not be taken to imply that localized CQD states are microscopically equivalent to chemical deep defects. Rather, it emphasizes that strong localization is undesirable for an intermediate band because it suppresses its collective electronic character. Non-radiative recombination processes themselves are not included in the present model.

Accordingly, the mere presence of a high density of electronic states within the host bandgap is not sufficient to constitute a useful intermediate band. The relevant states should remain sufficiently extended and mutually coupled to preserve the collective character of the IB, rather than evolving into a set of isolated, defect-like states. This consideration provides a direct physical motivation for analysing not only the spectral position and width of the intermediate band, but also the spatial extension of its eigenstates. It is precisely this latter aspect that motivates the state-resolved participation analysis adopted in the present work.

The problem is closely related to localization in disordered electronic systems. Disorder in on-site energies and hopping amplitudes can strongly modify the spatial extent of eigenstates and, under sufficiently strong conditions, lead to Anderson-type localization [64,65,66]. Real CQD ensembles naturally contain several microscopic sources of such disorder. Two particularly relevant ones are variations in QD size and position. Radius fluctuations alter the quantum-confinement energy and therefore introduce diagonal disorder in the effective QD energy levels. At the same time, they modify QD surface separations and wavefunction decay lengths and consequently perturb the tunnelling amplitudes. Positional fluctuations primarily modify the interdot distances and hence generate off-diagonal disorder through the exponential distance dependence of tunnelling. Previous studies have examined transport and connectivity in disordered CQD ensembles, including network-based descriptions [67]. However, the disorder-dependent spatial integrity of collective IB eigenstates in three-dimensional CQDM systems has received comparatively little attention.

Here, we investigate this problem using a single-orbital tight-binding model of a large three-dimensional ensemble of electronically coupled CQDs embedded in a perovskite-like matrix. The ordered reference structure is a dilated face-centered-cubic lattice, and radius and positional fluctuations are introduced explicitly and independently at the level of individual CQDs. In contrast with approaches in which structural dispersion appears only as a phenomenological optical broadening, disorder therefore enters directly into the microscopic Hamiltonian through both on-site energies and interdot hopping amplitudes.

For each disorder realization, the Hamiltonian is diagonalized and the spatial extension of the IB eigenstates is quantified through the inverse participation ratio (IPR) and the normalized participation ratio. This state-resolved description enables us to distinguish spectral broadening from the actual loss of spatially extended states. We further introduce a contiguous, participation-defined spectral core and characterize it through its mean participation, its relative energy width, and a combined delocalization–robustness descriptor. In addition, a finite-size analysis is used to examine how the effective number of CQDs participating in this core scales with system size.

The novelty of the present work therefore does not lie in establishing that QD size controls confined energy levels—a dependence already well known and quantitatively analysed in Ref. [62]—but in determining how fluctuations in QD size and position affect the collective eigenstate structure of a coupled CQD-derived intermediate band. Specifically, the present approach (i) introduces radius and positional disorder microscopically in a large three-dimensional CQD ensemble; (ii) connects these structural fluctuations with diagonal and off-diagonal disorder in the effective Hamiltonian; (iii) resolves the localization response as a function of eigenenergy; (iv) distinguishes the total IB width from the energy interval actually occupied by comparatively extended states; and (v) directly compares the sensitivity of the intermediate band to radius and positional disorder over a two-dimensional disorder map.

The calculations reveal that disorder degrades the intermediate band in an energy-selective manner: states close to the spectral edges lose spatial participation before the most extended states in the central region. Consequently, the total IB width alone can overestimate the portion of the band that remains spatially useful. Within the adopted parameterization, radius fluctuations produce a substantially stronger degradation than moderate positional fluctuations, identifying CQD size uniformity as a particularly restrictive control variable for preserving a high-participation IB core. These conclusions refer to the spatial structure of the effective one-particle eigenstates and are not intended as direct predictions of optical absorption, carrier mobility, or photovoltaic conversion efficiency.

The remainder of this paper is organized as follows. Section 2 presents the model and its underlying assumptions. Section 3 defines the participation metrics, Section 4 presents the numerical results, and Section 5 discusses their interpretation and limitations. Finally, Section 6 summarizes the main conclusions.

## 2. Model and Assumptions

### 2.1. Physical Scope and Modelling Assumptions

We consider an intermediate band (IB) material formed by electronically coupled PbS colloidal quantum dots (CQDs) embedded in a halide perovskite matrix. We represent each CQD by a single effective orbital associated with the confined electronic level from which the intermediate band (IB) arises. Note that the perovskite matrix is not explicitly included in the Hilbert space. Instead, it mediates the electronic coupling between neighboring CQDs and acts as a finite tunnelling barrier.

The purpose of our model is not to reproduce the complete electronic structure of either PbS or the perovskite host. Rather, it provides an effective one-particle description designed to isolate the influence of structural disorder on IB formation and on the spatial extension of its eigenstates. Spin degeneracy, Coulomb charging, excitonic effects, surface-trap manifolds, and the explicit valence and conduction bands of the host material are therefore neglected. For reasons that will become clear later, we will return to these arguments in more detail after introducing some necessary concepts.

We assume that the ordered reference system is a dilated face-centered-cubic (FCC) lattice. This geometry provides an isotropic arrangement with twelve nearest neighbors per CQD and constitutes a natural ordered reference for approximately spherical, non-overlapping inclusions. Unlike a close-packed FCC structure, the lattice is deliberately dilated so that neighboring CQDs remain separated by the perovskite matrix.

### 2.2. The Dilated FCC Reference Geometry

The reference geometry is defined directly from the mean CQD radius $R_{0}$ and the CQD volume fraction $f_{\mathrm{CQD}}$. The volume and number density of the mean CQD are

(1) $$V_{\mathrm{QD}}^{\left(0\right)}=\frac{4\pi R_{0}^{3}}{3},\;n_{\mathrm{CQD}}=\frac{f_{\mathrm{CQD}}}{V_{\mathrm{QD}}^{\left(0\right)}}.$$

Since an FCC unit cell contains four CQDs,

(2) $$n_{\mathrm{CQD}}=\frac{4}{a_{\mathrm{FCC}}^{3}},$$

the lattice constant is

(3) $$a_{\mathrm{FCC}}={\left(\frac{16\pi R_{0}^{3}}{3f_{\mathrm{CQD}}}\right)}^{1/3}.$$

The corresponding nearest-neighbor distance and ideal surface-to-surface separation are

(4) $$d_{\mathrm{nn}}^{\mathrm{FCC}}=\frac{a_{\mathrm{FCC}}}{\sqrt{2}},\;s_{0}^{\mathrm{FCC}}=d_{\mathrm{nn}}^{\mathrm{FCC}}-2R_{0}.$$

For instance, the reference values $R_{0}=2.0\;\mathrm{nm}$ and $f_{\mathrm{CQD}}=0.14$, which lies within the dense PbS-QD loading range reported for PbS/halide–perovskite intermediate-band structures [54], lead to

(5) $$a_{\mathrm{FCC}}≃9.86\;\mathrm{nm},\;d_{\mathrm{nn}}^{\mathrm{FCC}}≃6.97\;\mathrm{nm},\;s_{0}^{\mathrm{FCC}}≃2.97\;\mathrm{nm}.$$

Thus, the CQDs form a dense electronically coupled ensemble while remaining physically separated by the matrix.

### 2.3. Structural Disorder

As previously mentioned, we consider two sources or channels of structural disorder: CQD radius dispersion and positional fluctuations about the ideal FCC sites. Because unbounded Gaussian distributions may generate arbitrarily large CQDs or displacements, the stochastic variables are taken from bounded distributions.

Gaussian distributions are commonly used to represent size and positional fluctuations in QD ensembles [68,69,70]. Here, the Gaussian variables are additionally truncated in order to exclude the arbitrarily large size and positional deviations allowed by an unbounded normal distribution, which would be incompatible with the intended weak-to-moderate structural-disorder regime.

The use of a Gaussian distribution is motivated by experimentally observed QD size distributions and by previous theoretical treatments of size disorder in coupled QD arrays [68]. We use a truncated Gaussian rather than an unbounded normal distribution to prevent rare realizations with physically unrealistic QD radii. Thus, we write the radius of CQD *i* as

(6) $$R_{i}=R_{0}\left(1+\sigma_{R}\xi_{i}\right),$$

where $\sigma_{R}$ is a nominal dimensionless radius-disorder amplitude and

(7) $$\xi_{i}∼N_{\mathrm{tr}}\left(0,1;-k_{R},k_{R}\right)$$

is a standard normal random variable truncated to the interval $\left[-k_{R},k_{R}\right]$. Consequently,

(8) $$R_{0}\left(1-k_{R}\sigma_{R}\right)\leq R_{i}\leq R_{0}\left(1+k_{R}\sigma_{R}\right).$$

Truncation excludes physically unrealistic radius values. Because the distribution is truncated, $\sigma_{R}$ is a scale parameter rather than the realized standard deviation of $\left(R_{i}-R_{0}\right)/R_{0}$; the latter equals $\sigma_{R}\sqrt{\mathrm{Var}(\xi )}\approx 0.880\sigma_{R}<\sigma_{R}$ for $k_{R}=2$, even before the application of the geometrical acceptance condition.

Positional fluctuations around ideal lattice sites are represented by isotropic Gaussian displacements, consistently with previous treatments of positional disorder in QD arrays [69,70]. In the present model, the displacement magnitude is bounded to exclude unrealistically large deviations from the reference FCC lattice. This is the reason why we introduce the positional disorder as a bounded displacement from each ideal FCC site as

(9) $$r_{i}=r_{i}^{\left(0\right)}+\Delta r_{i},\;\Delta r_{i}=\sigma_{P}d_{\mathrm{nn}}^{\mathrm{FCC}}\eta_{i},$$

where $\eta_{i}$ is an isotropic three-dimensional Gaussian vector conditioned to fulfill

(10) $$\left|\eta_{i}\right|\leq k_{P}.$$

Thereby, the maximum displacement is

(11) $$\left|\Delta r_{i}\right|\leq k_{P}\sigma_{P}d_{\mathrm{nn}}^{\mathrm{FCC}}.$$

This definition gives $\sigma_{P}$ a nominal geometrical interpretation relative to the ordered nearest-neighbor distance. It is not the root-mean-square displacement normalized by $d_{\mathrm{nn}}^{\mathrm{FCC}}$, for two distinct reasons. First, for an isotropic three-dimensional displacement, the total RMS magnitude exceeds the per-component scale by a factor $\sqrt{3}$. Second, the conditioning $\left|\eta_{i}\right|\leq k_{P}$ further reduces the realized dispersion, by a factor of approximately 0.781 for $k_{P}=2$. The combination of both effects yields $\delta_{P,\mathrm{RMS}}\approx 1.35\sigma_{P}$. Similarly, the truncation of the radial variable at $k_{R}=2$ reduces its realized dispersion to $\sigma_{R}\sqrt{\mathrm{Var}(\xi )}\approx 0.880\sigma_{R}$. Accordingly, equal numerical values of $\sigma_{R}$ and $\sigma_{P}$ do not represent statistically equivalent disorder variances, and the comparison between the two channels reported below refers specifically to the adopted parameterization.

For every generated realization, we compute the distance between two CQDs using the minimum-image convention,

(12) $$d_{ij}={\left|r_{i}-r_{j}\right|}_{\mathrm{PBC}},$$

the corresponding surface-to-surface separation being

(13) $$s_{ij}=d_{ij}-R_{i}-R_{j}.$$

Only configurations fulfilling the hard geometrical constraint

(14) $$\min_{i<j}s_{ij}\geq s_{\min}$$

are accepted. Configurations violating Equation (14) are discarded and regenerated. This condition ensures that different CQDs remain separated by a finite matrix barrier and prevents touching or overlapping inclusions from being artificially treated by the tight-binding model.

With the bounded distributions and the parameter ranges adopted in this work, the minimum surface separation attainable in the most unfavourable configuration is approximately 1.18 nm at $\left(\sigma_{R},\sigma_{P}\right)=\left(0.05,0.05\right)$, well above $s_{\min}=0.5$ nm. As will be shown, no realization was rejected in our simulations, and the constraint of Equation (14) therefore acts as a safeguard for stronger-disorder extensions of the model rather than as an active filter that would bias the realized disorder statistics.

The interaction graph is constructed geometrically by connecting QD pairs that satisfy

(15) $$\left(i,j\right)\in E\;⟺\;d_{ij}\leq r_{c},\;r_{c}=\gamma_{c}d_{\mathrm{nn}}^{\mathrm{FCC}},$$

where $\gamma_{c}$ is selected to recover the twelve nearest neighbors in the ordered limit.

### 2.4. Effective Tight-Binding Hamiltonian

With this in mind, the CQD-derived IB is described by the single-orbital tight-binding Hamiltonian

(16) $$H=\sum_{i}\varepsilon_{i}|i\rangle \langle i|+\sum_{(i,j)\in E}t_{ij}\left(|i\rangle \langle j|+|j\rangle \langle i|\right),$$

where |i〉 denotes the effective IB orbital localized on CQD *i*, $\varepsilon_{i}$ is its on-site energy, and $t_{ij}$ is the matrix-mediated tunnelling energy.

The on-site energy is separated into an average IB position and a radius-dependent contribution,

(17) $$\varepsilon_{i}=\varepsilon_{\mathrm{IB}}^{\left(0\right)}+A_{R}\left[{\left(\frac{R_{0}}{R_{i}}\right)}^{2}-1\right].$$

Since a uniform energy shift does not modify the eigenvectors, the absolute value of $\varepsilon_{\mathrm{IB}}^{\left(0\right)}$ is irrelevant when only localization and relative spectral properties are considered.

Note that the radius dispersion not only introduces diagonal energy disorder but also modifies the tunnelling values. In this respect, the tunnelling decay length associated with CQD *i* can be modelled as

(18) $$\lambda_{i}=\lambda_{0}{\left(\frac{R_{i}}{R_{0}}\right)}^{\beta_{\lambda }},\;\lambda_{ij}=\frac{\lambda_{i}+\lambda_{j}}{2}.$$

With this approach, the tunnelling energy between CQDs *i* and *j* is then

(19) $$t_{ij}=t_{0}\exp\left[-\frac{s_{ij}-s_{0}^{\mathrm{FCC}}}{\lambda_{ij}}\right].$$

In the ordered limit, $R_{i}=R_{j}=R_{0}$ and $s_{ij}=s_{0}^{\mathrm{FCC}}$, so that $t_{ij}=t_{0}$. Increasing the separation reduces the coupling exponentially, whereas bringing two CQDs closer, without violating the hard-exclusion condition, increases it.

The construction method that we propose provides a controlled interpolation between an ideal FCC reference and a structurally disordered CQD-in-matrix system. Radius disorder modifies both on-site energies (Equation (17)) and hopping energies (Equation (19)), whereas positional disorder acts mainly through surface-to-surface separation. The analysis below therefore compares certain metrics that we will define later with two descriptive disorder scales, while recognizing that the diagonal and off-diagonal Hamiltonian disorder components are not independently controlled by the present disorder map.

For each realization, the on-site-energy dispersion is

(20) $$\sigma_{E}={\left[\frac{1}{N}\sum_{i=1}^{N}{\left(\varepsilon_{i}-\langle \varepsilon \rangle \right)}^{2}\right]}^{1/2},$$

and the relative tunnelling-magnitude dispersion is

(21) $$\frac{\sigma_{t}}{\langle |t|\rangle },\;\sigma_{t}={\left[\frac{1}{\left|E\right|}\sum_{(i,j)\in E}{\left(|t_{ij}|-\langle |t|\rangle \right)}^{2}\right]}^{1/2}.$$

We have mentioned above that the goal of our model is not to generate the complete electronic structure of a PbS quantum dot or the perovskite matrix. Our approach should be understood as a projection onto a single set of electronic states, rather than a literal, single-level description of a lead-chalcogenide nanocrystal. Actual PbS and PbSe CQDs exhibit valley-derived state sets and multi-state orbitals closely spaced together, and both experiments and atomistic calculations show a richer level structure than that suggested by a single-orbital model [71,72].

However, in this respect, a single-manifold reduction is more appropriate when the separation $\Delta_{\mathrm{orb}}$ to the nearest neglected CQD manifold remains larger than both the characteristic interdot-coupling and disorder energy scales [73],

(22) $$\Delta_{\mathrm{orb}}\gg \langle |t|\rangle ,\;\sigma_{E},\;\sigma_{t}.$$

In this regime, the neglected manifolds primarily renormalize the effective on-site energies and hopping amplitudes. If orbital splittings become comparable to any of these energy scales, however, structural disorder and interdot coupling can mix different orbital and valley states. A multi-orbital Hamiltonian would then be required, with orbital-dependent on-site energies, hopping matrices, and disorder-induced inter-manifold hybridization [73]. Such processes could quantitatively modify the participation spectrum, the retained spectral core, and the location of the disorder crossover. The present results therefore isolate the single-particle structural-disorder response of one effective CQD-derived manifold.

We have also stated before that the perovskite matrix is not explicitly included in the Hilbert space. Isolation of the CQD-derived manifold from the host valence and conduction bands is assumed rather than tested in the present model. Its tunnelling barrier and the CQD–matrix interface enter through the effective hopping scale $t_{0}$, the decay length $\lambda_{0}$, and the distance dependence of $t_{ij}$. Surface chemistry, ligands, dielectric mismatch, local band offsets, and interface dipoles can alter these effective quantities, but are not resolved microscopically here. This limitation is important when transferring the numerical crossover to a particular CQD/matrix chemistry.

Furthermore, note that spin degeneracy, Coulomb charging, long-range Coulomb interactions, many-body correlations, excitonic effects, electron–phonon coupling, and surface-trap manifolds are not included in the model we propose. The calculated participation descriptors therefore characterize the spatial structure of a one-particle effective manifold and should not be interpreted as a complete finite-temperature transport or device model.

## 3. State-Resolved Participation Metrics

For each disorder realization, the Hamiltonian can be diagonalized as

(23) $$H|\psi_{n}\rangle =E_{n}|\psi_{n}\rangle ,$$

with normalized eigenstates,

(24) $$\sum_{i=1}^{N_{\mathrm{IB}}}{\left|\psi_{n}\left(i\right)\right|}^{2}=1,$$

$N_{\mathrm{IB}}$ being the number of states assigned to the IB. In the present one-orbital model, $N_{\mathrm{IB}}=N$.

These IB states can be ordered by energy,

(25) $$E_{1}\leq E_{2}\leq \cdots \leq E_{N}.$$

With all this in mind, the inverse participation ratio (IPR) and the corresponding effective participation number are

(26) $${\mathrm{IPR}}_{n}=\sum_{i=1}^{N}{\left|\psi_{n}\left(i\right)\right|}^{4},\;N_{\mathrm{eff},n}=\frac{1}{{\mathrm{IPR}}_{n}},$$

the normalized participation ratio being

(27) $$P_{n}=\frac{N_{\mathrm{eff},n}}{N}=\frac{1}{N\;{\mathrm{IPR}}_{n}}.$$

It fulfils $1/N\leq P_{n}\leq 1$. A value $P_{n}=p$ corresponds to an effective participation number pN. It should not be interpreted as the literal fraction of sites on which the wavefunction is non-zero.

We define the largest normalized participation within the intermediate band as

(28) $$\Pi_{\max}=\max_{1\leq n\leq N_{\mathrm{IB}}}P_{n}.$$

For a threshold q∈(0,1), let $n_{★}$ be an index at which $\Pi_{\max}$ is attained. If several states share the maximum, the one closest to the spectral midpoint $(E_{1}+E_{N_{\mathrm{IB}}})/2$ is selected. The lower and upper indices of the contiguous threshold-defined core are

(29) $$n_{-}\left(q\right)=\min\;\left\{n\leq n_{★}:P_{m}\geq q\Pi_{\max}\;\mathrm{for}\;\mathrm{all}\;m=n,\ldots ,n_{★}\right\},$$

(30) $$n_{+}\left(q\right)=\max\;\left\{n\geq n_{★}:P_{m}\geq q\Pi_{\max}\;\mathrm{for}\;\mathrm{all}\;m=n_{★},\ldots ,n\right\}.$$

The spectral core is the maximal contiguous sequence

(31) $$C_{q}=\left\{n_{-}\left(q\right),n_{-}\left(q\right)+1,\ldots ,n_{+}\left(q\right)\right\}.$$

This definition avoids spanning disconnected groups of states that happen to exceed the threshold.

The extended core width is

(32) $$\Delta E_{q}=E_{n_{+}\left(q\right)}-E_{n_{-}\left(q\right)}$$

Thus, the normalized core-width descriptor is

(33) $$\eta_{q}=\frac{\Delta E_{q}}{W_{\mathrm{IB}}},$$

where $W_{\mathrm{IB}}$ is the total spectral width of the IB, which is defined, for each realization, as

(34) $$W_{\mathrm{IB}}=E_{N_{\mathrm{IB}}}-E_{1}.$$

The mean participation within the core is

(35) $$\Pi_{\mathrm{core}}^{\left(q\right)}=\frac{1}{|C_{q}|}\sum_{n\in C_{q}}P_{n},\;\left|C_{q}\right|=n_{+}\left(q\right)-n_{-}\left(q\right)+1.$$

Finally, the combined delocalization–robustness descriptor is

(36) $$A_{\mathrm{IB}}^{\left(q\right)}=\Pi_{\mathrm{core}}^{\left(q\right)}\eta_{q}.$$

Throughout this work, we use the operational threshold q=0.5 and write

(37) $$\eta_{0.5}=\frac{\Delta E_{0.5}}{W_{\mathrm{IB}}},\;A_{\mathrm{IB}}^{\left(0.5\right)}=\Pi_{\mathrm{core}}^{\left(0.5\right)}\cdot \eta_{0.5}.$$

Please note that the choice q=0.5 is an operational convention analogous to a half-maximum construction. It is not a critical localization threshold. Likewise, $A_{\mathrm{IB}}^{\left(0.5\right)}$ is a bounded summary of spatial participation and spectral support, not a direct transport coefficient or device-efficiency metric.

## 4. Results

### 4.1. Computational Workflow

Our computational procedure is as follows:

1. Generate the ideal FCC positions for a finite periodic supercell.
2. Draw random quantum-dot radii $R_{i}$ and displaced positions $r_{i}$.
3. Reject the realization if the minimum surface separation violates $s_{\min}$.
4. Construct the neighbor graph using the cutoff $r_{c}=\gamma_{c}d_{\mathrm{nn}}$.
5. Compute on-site energies $\epsilon_{i}$ and hopping amplitudes $t_{ij}$.
6. Assemble the tight-binding Hamiltonian *H*.
7. Diagonalize *H* exactly for each disorder realization.
8. Compute state-resolved participation metrics $P_{n}$, $N_{\mathrm{eff},n}$, and descriptors or metrics.
9. Aggregate metrics over disorder realizations.
10. Perform finite-size scaling over multiple system sizes.

The main simulation parameters are listed in Table 1. As we will describe in more detail later, the specific values of the parameters $t_{0}$, $\lambda_{0}$, and $A_{R}$ are intended as effective reference scales rather than as a unique parametrization of a specific PbS/perovskite interface. Their values are selected to generate an IB width and disorder-energy scale representative of the weak-to-moderate coupling regime considered here. Consequently, the numerical crossover values reported below should be interpreted within this baseline parametrization.

The following paragraphs justify the numerical values that we have given to the parameters involved in our simulations.

The adopted $R_{0}=2\;\mathrm{nm}$ corresponds to the 4nm PbS QDs used in dense PbS/${\mathrm{CH}}_{3}{\mathrm{NH}}_{3}{\mathrm{PbBr}}_{3}$ IB structures by Hosokawa et al. [54]. Similarly, the selected value $f_{\mathrm{CQD}}=0.14$ lies within the experimentally demonstrated CQD volume fraction of 11–14% in [54]. The exponential tunnelling form is motivated by the strong barrier-length dependence of wavefunction overlap in nanocrystal solids [74] and by calculations showing that dot–matrix band offsets and interface chemistry strongly affect transport in quantum-dot-in-perovskite solids [75].

The selected $t_{0}=5\;\mathrm{meV}$ and $\lambda_{0}=1\;\mathrm{nm}$ therefore represent a weak-to-moderate effective coupling scale, not universal material constants. We justify the values used throughout this work in the following two paragraphs.

The reference tunnelling parameters can be related to microscopic coupling scales reported for nanocrystal solids. Erwin and Efros [75] calculated the electronic miniband width of PbS quantum dots embedded in a CsPbI_3_ perovskite matrix and showed that it varies over several orders of magnitude with the dot–matrix band offset and matrix thickness. In the weak-barrier regime, the resulting electronic energy scales lie in the meV to tens-of-meV range. Direct first-principles calculations for PbS nanocrystal solids also produce electronic couplings between quantum dots in the range of sub-meV to meV, with strong variations according to the orientation and the separation of the facets [76]. We therefore use $t_{0}=5\;\mathrm{meV}$ as a representative few-meV nearest-neighbor coupling scale rather than as a uniquely fitted microscopic hopping integral.

The exponential dependence adopted for the hopping, $t_{ij}\propto \exp\left[-\left(s_{ij}-s_{0}\right)/\lambda_{ij}\right]$, is consistent with the evanescent decay of quantum-confined wavefunctions through an intervening potential barrier [74,75]. The penetration length is

(38) $$\lambda_{\mathrm{pen}}=\frac{\hbar }{\sqrt{2m_{B}U_{B}}},$$

where $m_{B}$ and $U_{B}$ are the effective mass and barrier height in the barrier material or matrix, respectively. Erwin and Efros used a CsPbI_3_ band-edge mass $m_{B}=0.32m_{0}$ for PbS/CsPbI_3_ [75]. For an effective barrier in the range $U_{B}≃0.1$–0.2eV, this gives $\lambda_{\mathrm{pen}}≃0.8$–1.1nm. Thus, $\lambda_{0}=1\;\mathrm{nm}$ represents a physically plausible effective decay length for a relatively low-barrier QD–perovskite interface. It should not be interpreted as the decay length of an organic ligand barrier, which can be considerably shorter.

The effective radial sensitivity $A_{R}$ can be related to the experimentally established size dependence of PbS CQD energy levels. Moreels et al. reported the empirical sizing relation [77]

(39) $$E_{G}\left(D\right)=0.41+\frac{1}{0.0252D^{2}+0.283D},$$

with $E_{G}$ in eV and the CQD diameter *D* in nm. At the reference diameter $D_{0}=2R_{0}=4$ nm, this relation gives a local logarithmic sensitivity

(40) $${\left.\frac{\partial E_{G}}{\partial \ln D}\right|}_{D_{0}}≃-0.82\;\mathrm{eV}.$$

Since PbS has similar electron and hole effective masses, the confinement-induced shift of an individual band-edge level is expected, to first approximation, to account for roughly one half of the optical gap shift. This gives a characteristic single-level sensitivity of approximately 0.41 eV per unit fractional size change. In the present parameterization, $\delta \epsilon ≃-2A_{R}\;\delta R/R_{0}$ for small radius fluctuations, so that this experimental size dependence corresponds to $A_{R}≃0.20$ eV. We therefore use $A_{R}=200$ meV as a physically motivated effective reference value rather than as a uniquely fitted PbS/perovskite interface parameter.

The radius dependence of the tunnelling decay length is described phenomenologically through Equation (18). The negative sign of $\beta_{\lambda }$ is consistent with the confinement model adopted here: increasing the CQD radius lowers the confined electron level and therefore increases its effective barrier relative to a fixed host conduction-band edge, reducing the penetration length into the matrix. Since

(41) $$\lambda_{\mathrm{pen}}\propto U_{B}^{-1/2},$$

linearization around $R_{0}$ leads to an effective logarithmic exponent

(42) $$\beta_{\lambda ,\mathrm{eff}}=\frac{d\ln\lambda }{d\ln R}≃-\frac{A_{R}}{U_{B}}.$$

With $A_{R}=0.20\;\mathrm{eV}$ and the representative barrier range $U_{B}≃0.1$–0.2eV considered above, this corresponds to $\beta_{\lambda ,\mathrm{eff}}∼-2\;\mathrm{to}\;-1.$ We therefore adopt $\beta_{\lambda }=-1$ as a conservative baseline value rather than as a uniquely fitted material parameter.

As $\sigma_{R}$ and $\sigma_{P}$ are scale parameters of bounded random variables, we also report on the disorder that occurs in our computational work. Regarding this, for each realization, we define the quantities that follow.

The first one, $\delta_{R}^{\mathrm{real}}$, is the relative radius dispersion, defined as the standard deviation (SD) of the CQD radii normalized by their sample mean radius $\bar{R}$:

(43) $$\delta_{R}^{\mathrm{real}}=\frac{\mathrm{SD}(R_{i})}{\bar{R}}.$$

Second, the positional disorder is characterized by the root-mean-square (RMS) displacement of the CQDs from their ideal FCC lattice sites, normalized by the ordered nearest-neighbor distance $d_{\mathrm{nn}}^{\mathrm{FCC}}$:

(44) $$\delta_{P,\mathrm{RMS}}^{\mathrm{real}}=\frac{1}{d_{\mathrm{nn}}^{\mathrm{FCC}}}\sqrt{\frac{1}{N}\sum_{i}{\left|\Delta r_{i}\right|}^{2}},$$

where $\Delta r_{i}=r_{i}-r_{i}^{\left(0\right)}$ is the displacement vector of CQD *i* relative to its ideal FCC position.

For the isotropic displacement distribution used in the model, the corresponding one-dimensional positional-disorder scale is defined as the RMS displacement per Cartesian component,

(45) $$\delta_{P,1D}^{\mathrm{real}}=\frac{\delta_{P,\mathrm{RMS}}^{\mathrm{real}}}{\sqrt{3}}.$$

Thus, $\delta_{P,1D}^{\mathrm{real}}$ provides a component-wise measure that is more directly comparable to a conventional standard deviation, whereas $\delta_{P,\mathrm{RMS}}^{\mathrm{real}}$ measures the total three-dimensional displacement magnitude.

In this regard, Table 2 lists the ensemble means for the pure radius- and pure positional-disorder channels or processes. The quoted uncertainties are the standard deviations across the 20 realizations.

For $\bar{R}\approx R_{0}=2$ nm with nominal amplitude 0.05, $\delta_{R}^{\mathrm{real}}=$ 4.418% corresponds to SD(R) =0.0884 nm, or equivalently to a diameter standard deviation of approximately 0.177 nm for $D_{0}\approx 4$ nm.

The largest realized radial dispersion is also in the few-percent range for which PbS-CQD transport has been reported to be strongly sensitive to monodispersity [78]. Note that this is an order-of-magnitude comparison rather than an identification of the two dispersity definitions.

### 4.2. Disorder Maps and Line Cuts

**Figure 2:**
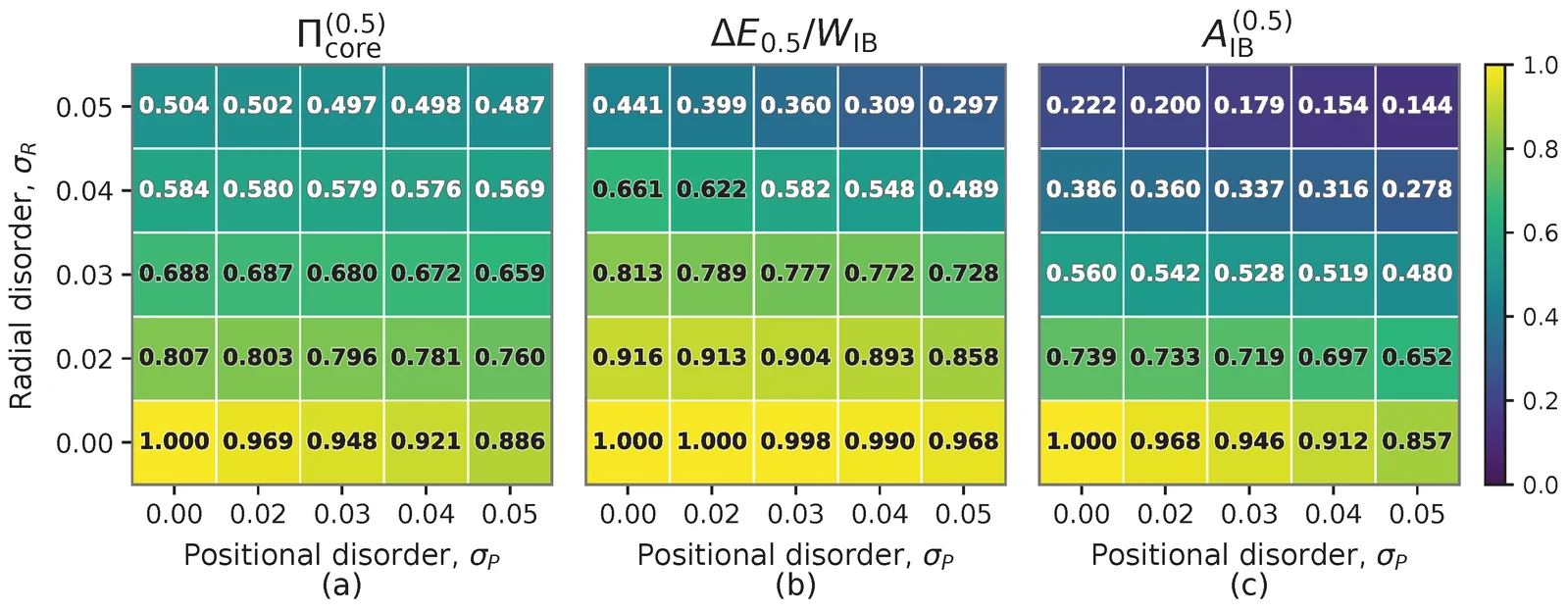
Ensemble-averaged descriptors as functions of nominal radius disorder $\sigma_{R}$ and nominal positional disorder $\sigma_{P}$: (**a**) core participation $\Pi_{\mathrm{core}}^{\left(0.5\right)}$; (**b**) core energy-width fraction $\eta_{0.5}=\Delta E_{0.5}/W_{\mathrm{IB}}$; and (**c**) combined robustness descriptor $A_{\mathrm{IB}}^{\left(0.5\right)}$. Equal numerical values of $\sigma_{R}$ and $\sigma_{P}$ do not represent equal statistical variances.

Figure 2 shows the three previously defined descriptors or metrics over the two-dimensional parameter map. The principal visual trend is the stronger variation along the nominal radius-disorder direction than along the nominal positional-disorder direction. Since $\sigma_{R}$ and $\sigma_{P}$ have different stochastic and geometrical definitions, comparisons at equal numerical values refer specifically to the adopted parameterization and not to equal disorder variances.

For the ordered reference, all three descriptors equal unity. With nominal positional disorder alone, $\Pi_{\mathrm{core}}^{\left(0.5\right)}$ decreases to approximately 0.886 at $\sigma_{P}=0.05$. Therefore, 0.886 means approximately 88.6% of the participation value of the ordered system. With nominal radius disorder alone, the same descriptor decreases more strongly, reaching approximately 0.504 at $\sigma_{R}=0.05$. When both amplitudes equal 0.05, it is approximately 0.487. Thus, within this parameter map, the additional effect of positional disorder on core participation is comparatively small once radius disorder is already large.

The core energy-width fraction shows a similar but more nonlinear response. At $\sigma_{R}=0$, it changes from 1.000 to approximately 0.968 as $\sigma_{P}$ increases to 0.05. Across the positional-disorder axis, the approximate ranges are

(46) $$\eta_{0.5}\approx \left\{\begin{array}{cc} 0.858–0.916, & \sigma_{R}=0.02,\\ 0.728–0.813, & \sigma_{R}=0.03,\\ 0.489–0.661, & \sigma_{R}=0.04,\\ 0.297–0.441, & \sigma_{R}=0.05. \end{array}\right.$$

The pronounced reduction between $\sigma_{R}=0.03$ and 0.05 identifies a model-dependent crossover in the retained energy span. Since $\eta_{0.5}$ is an energy-width fraction, these values should not be read as fractions of the total number of states.

The combined descriptor amplifies the simultaneous decrease in spatial participation and energy support. Positional disorder alone gives $A_{\mathrm{IB}}^{\left(0.5\right)}≃0.857$ at $\sigma_{P}=0.05$, whereas radius disorder alone gives $A_{\mathrm{IB}}^{\left(0.5\right)}≃0.222$ at $\sigma_{R}=0.05$. The combined case $\left(\sigma_{R},\sigma_{P}\right)=\left(0.05,0.05\right)$ gives approximately 0.144. Within the square $\sigma_{R}\leq 0.03$ and $\sigma_{P}\leq 0.03$, the minimum map value is approximately

(47) $$A_{\mathrm{IB}}^{\left(0.5\right)}≃0.528,$$

so the descriptor satisfies $A_{\mathrm{IB}}^{\left(0.5\right)}≳0.528$ over that sampled region.

We avoid referring to this region as a universal “safety surface” because its boundary depends on the selected parameters and threshold.

**Figure 3:**
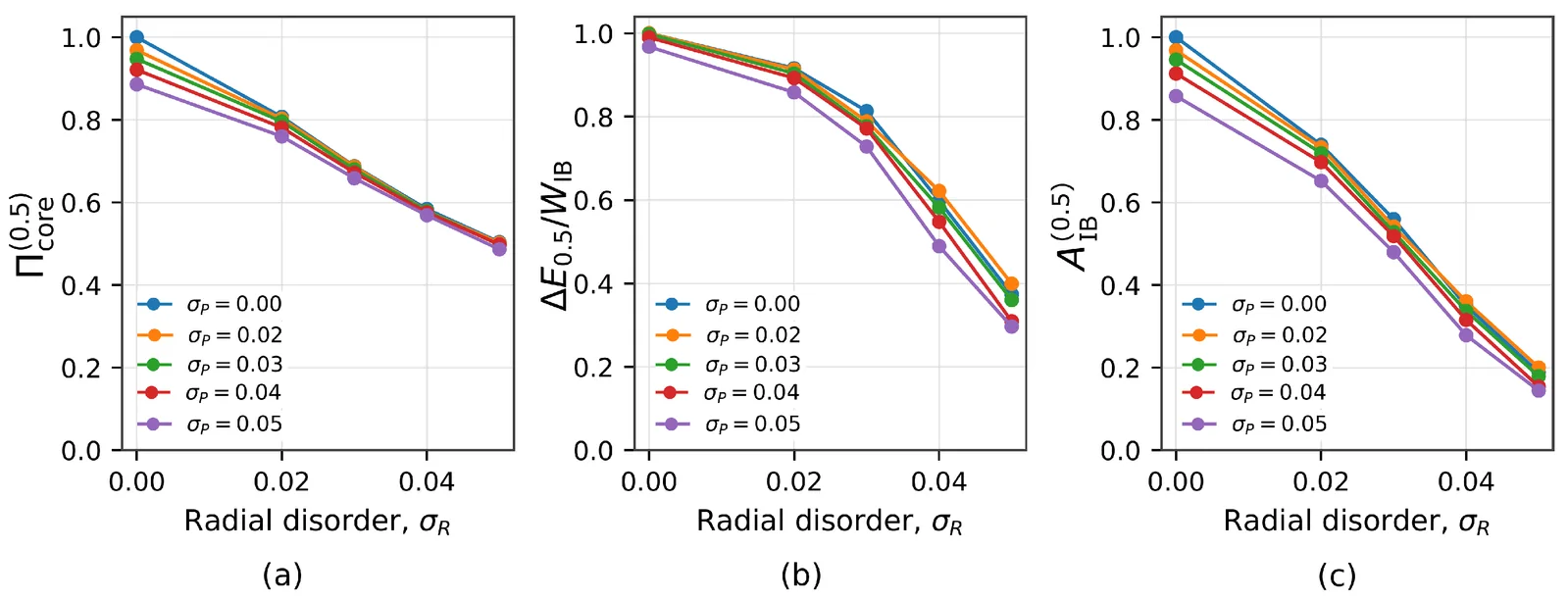
Line cuts versus nominal radius disorder for each nominal positional-disorder value: (**a**) core participation $\Pi_{\mathrm{core}}^{\left(0.5\right)}$; (**b**) core energy-width fraction $\eta_{0.5}$; and (**c**) combined descriptor $A_{\mathrm{IB}}^{\left(0.5\right)}$.

For illustrative purposes, Figure 3 represents the same data as line cuts versus $\sigma_{R}$.

Figure 3a shows an approximately monotonic decrease in $\Pi_{\mathrm{core}}^{\left(0.5\right)}$, while the curves for different $\sigma_{P}$ remain relatively close. Figure 3b shows that the core-width fraction changes more quickly beyond $\sigma_{R}≃0.03$. The approximate ranges of the combined descriptor are

(48) $$A_{\mathrm{IB}}^{\left(0.5\right)}\approx \left\{\begin{array}{cc} 0.65–0.74, & \sigma_{R}=0.02,\\ 0.48–0.56, & \sigma_{R}=0.03,\\ 0.28–0.39, & \sigma_{R}=0.04,\\ 0.14–0.22, & \sigma_{R}=0.05. \end{array}\right.$$

For the baseline parameter set, these values motivate the descriptive terms “comparatively robust” near $\sigma_{R}=0.02$, “crossover” around $\sigma_{R}=0.03$–0.04, and “strongly degraded” at the larger sampled radius-disorder amplitudes. Note again that these labels summarize the computed map and should *not* be considered as general manufacturing thresholds.

### 4.3. Energy-Resolved Relative Participation

**Figure 4:**
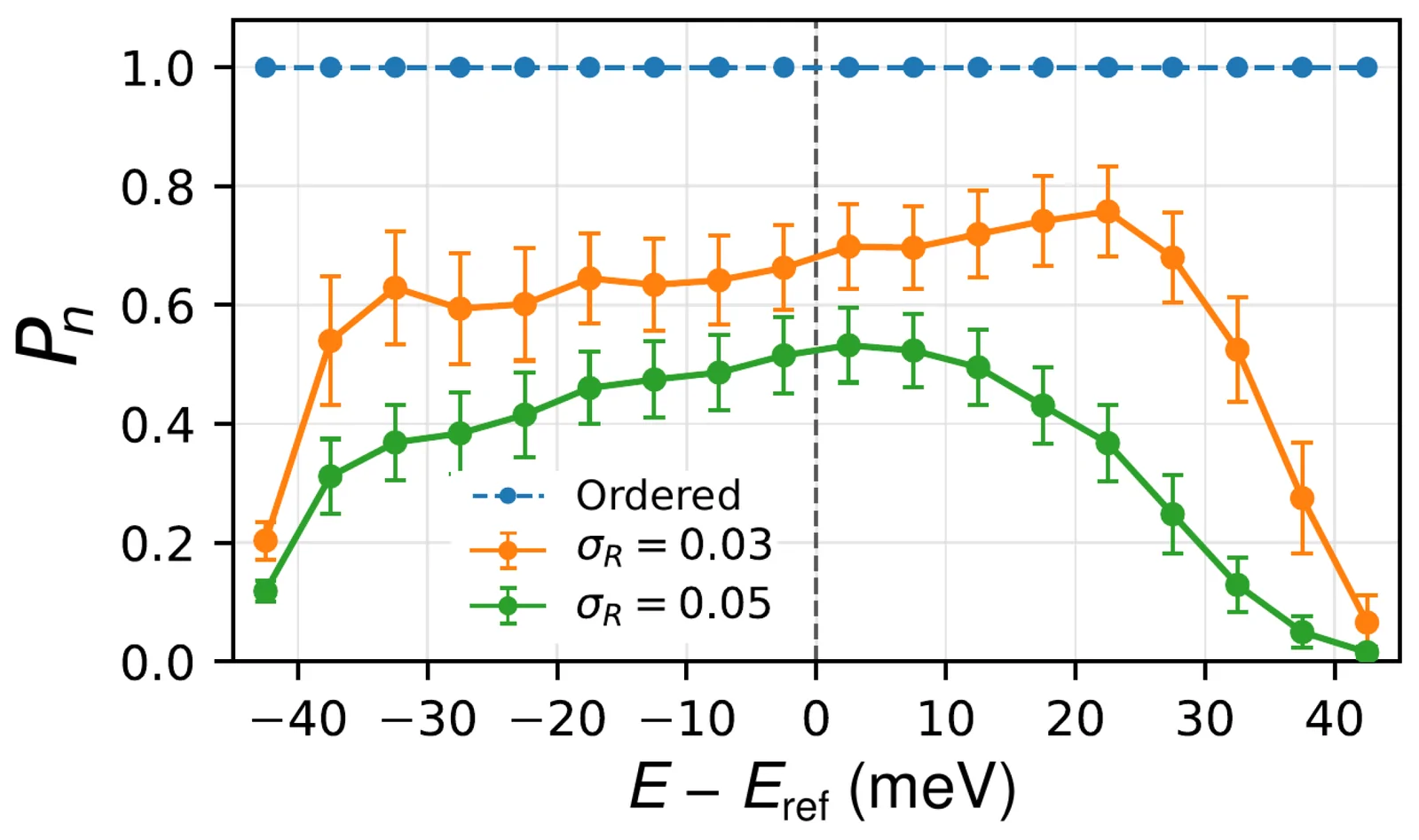
Ensemble-averaged normalized participation ratio $P_{n}$ as a function of the energy relative to a reference energy, $E-E_{\mathrm{ref}}$, for the ordered reference system and for radius-disorder amplitudes $\sigma_{R}=0.03$ and $\sigma_{R}=0.05$. $\sigma_{P}=0.00$. Error bars denote the standard deviation across disorder realizations.

Figure 4 shows the ensemble-averaged normalized participation ratio, $P_{n}$, as a function of the eigenenergy relative to a reference energy, $E-E_{\mathrm{ref}}$, for the ordered reference system and for two representative radius-disorder amplitudes, $\sigma_{R}=0.03$ and $\sigma_{R}=0.05$ (with $\sigma_{P}=0.00$). For graphical representation, each realization is referenced to its own spectral midpoint before ensemble averaging. That is, the energy axis is referenced to the spectral midpoint,

(49) $$E_{\mathrm{ref}}=\frac{E_{1}+E_{N_{\mathrm{IB}}}}{2}.$$

This choice provides a convenient zero-centered reference for comparing spectra with different total widths and is used exclusively for plotting. It does not enter the Hamiltonian, the eigenstate calculation, or the participation analysis, and it should not be interpreted as a self-consistent Fermi level or device chemical potential. Importantly, $E_{\mathrm{ref}}$ does not generally coincide with the isolated-QD reference level $\varepsilon_{\mathrm{IB}}^{\left(0\right)}$ shown in Figure 1c. For the ordered dilated-FCC lattice with twelve nearest neighbors and $t_{ij}=t_{0}>0$, the tight-binding band spans

(50) $$\left[\varepsilon_{\mathrm{IB}}^{\left(0\right)}-4t_{0},\;\varepsilon_{\mathrm{IB}}^{\left(0\right)}+12t_{0}\right],$$

so that its spectral midpoint is

(51) $$E_{\mathrm{ref}}=\varepsilon_{\mathrm{IB}}^{\left(0\right)}+4t_{0}.$$

A rigid shift of the energy origin does not modify any calculated eigenstates or participation quantities. The robustness of the observed spectral asymmetry is assessed below using a rank-based descriptor that is invariant under such rigid energy shifts.

In our model, a value $P_{n}=1$ corresponds to a state extended over the full system. More generally, $P_{n}=p$ implies an effective participation number $N_{\mathrm{eff},n}=pN$. It does not imply that the wavefunction vanishes on the remaining sites.

Figure 4 shows that, for the ordered system, the participation ratio remains essentially constant, with $P_{n}=1$ throughout the spectrum. Using the analytical ordered value $P_{n}=1$ avoids ambiguities associated with the arbitrary choice of eigenvectors within degenerate subspaces of the ordered Hamiltonian.

Additionally, Figure 4 also shows that, for moderate radius disorder, $\sigma_{R}=0.03$, the loss of spatial extension is strongly energy dependent. Near the spectral midpoint, the participation ratio is $P_{n}≃0.68–0.70$, so the corresponding states effectively involve about 70% of the quantum dots. The maximum participation, $P_{n}≃0.74–0.76$, occurs roughly 15–22meV above $E_{\mathrm{ref}}$. In contrast, the participation decreases towards the spectral edges: $P_{n}≃0.20$ near −40meV, $P_{n}≃0.28$ near +35meV, and it approaches zero close to the upper band edge at approximately +42meV.

The same energy-selective trend becomes more pronounced for $\sigma_{R}=0.05$. Around $E_{\mathrm{ref}}$, $P_{n}≃0.52–0.53$, and the maximum value remains close to 0.53. Thus, even the most extended states in this case effectively involve only about half of the quantum dots. Near the lower spectral edge, $P_{n}≃0.12$ at approximately −40meV. On the positive-energy side, $P_{n}$ decreases from about 0.37 to 0.13 between +20 and +30meV and becomes nearly zero at the upper spectral edge.

These results show that radius disorder does not produce a uniform downward shift of the participation curve. Instead, increasing $\sigma_{R}$ produces two simultaneous effects: it reduces the maximum participation of the central states and narrows the energy interval over which comparatively extended states persist. Consequently, the total IB width is not, by itself, a sufficient indicator of the spatial quality of the band. States near the spectral edges lose participation first, whereas a more extended central region survives over a broader disorder range.

Overall, the ordered reference exhibits fully extended states, whereas radius disorder first suppresses the participation of states near the band edges and then progressively reduces both the participation and the energy span of the central extended region. The case $\sigma_{R}=0.03$ retains a moderately extended spectral core, while $\sigma_{R}=0.05$ corresponds to a substantially degraded intermediate band. Although states near $E_{\mathrm{ref}}$ remain among the most extended in both disordered cases, $P_{n}$ is a structural eigenstate descriptor and should not be interpreted as a direct measure of conductivity, carrier mobility, recombination, or photovoltaic efficiency.

Aiming to analyze the origin and robustness of the spectral asymmetry, including whether it is robust across disorder realizations, we have considered the following analysis.

First, we introduced an asymmetry measure $S_{P}$ that does not need an energy origin. The eigenstates are ordered by energy and those states at symmetric ranks in the lower and upper halves of the spectrum are compared:

(52) $$S_{P}=\frac{\sum_{m=1}^{N/2}\left[P_{N/2+m}-P_{N/2+1-m}\right]}{\sum_{m=1}^{N/2}\left[P_{N/2+m}+P_{N/2+1-m}\right]}.$$

A negative value means that, on average over the whole spectrum, the higher-energy half has lower participation than the lower-energy half. Because this definition depends only on spectral rank, it is invariant under any rigid shift of the energy zero.

For 20 independent realizations, we have obtained $S_{P}=-0.179\pm 0.005$ and $S_{P}=-0.377\pm 0.011$ for $\sigma_{R}=0.03$ and $\sigma_{R}=0.05$, respectively. The uncertainties are the standard deviations across the 20 independent realizations. The sign of $S_{P}$ has been found to be negative in all 20 realizations for both disorder amplitudes. The spectral asymmetry is therefore systematic within the sampled ensemble rather than the result of a small number of atypical realizations.

Second, as a complementary check of the spectral asymmetry, we also compare the participation values at energies symmetrically located with respect to the graphical reference energy $E_{\mathrm{ref}}$. For each pair of energy offsets $\pm \Delta E_{k}$, we define the normalized mirror difference and average it over the *M* available symmetric pairs,

(53) $$S_{\mathrm{mir}}\left(E_{\mathrm{ref}}\right)=\frac{1}{M}\sum_{k=1}^{M}\frac{P\left(E_{\mathrm{ref}}+\Delta E_{k}\right)-P\left(E_{\mathrm{ref}}-\Delta E_{k}\right)}{P\left(E_{\mathrm{ref}}+\Delta E_{k}\right)+P\left(E_{\mathrm{ref}}-\Delta E_{k}\right)}.$$

A negative value suggests, on average, lower participation on the higher-energy side of the reference level. Applying Equation (53) to the ensemble-averaged curves in Figure 4 leads to approximately $S_{\mathrm{mir}}≃-0.058$ for $\sigma_{R}=0.03$ and $S_{\mathrm{mir}}≃-0.247$ for $\sigma_{R}=0.05$. Thus, the mirror comparison independently confirms the qualitative asymmetry visible in Figure 4, particularly for the larger radius-disorder amplitude. These values are intended as a descriptive measure of the ensemble-averaged spectral profile. The realization-to-realization robustness of the asymmetry is instead quantified by the rank-based descriptor $S_{P}$ above.

Unlike $S_{P}$, the mirror difference $S_{\mathrm{mir}}$ is defined with respect to a fixed energy origin and is therefore not invariant under a shift of that origin. It quantifies the asymmetry of the ensemble-averaged participation profile about the adopted graphical reference and is reported here only as a descriptive complement. The origin-independent statement about the asymmetry is the one provided by $S_{P}$.

Finally, we would like to make one last comment on the asymmetry obtained. An additional structural contribution is provided by the underlying FCC tight-binding dispersion itself. The nearest-neighbor FCC lattice is non-bipartite and therefore does not exhibit the particle–hole symmetry that would enforce a spectrum symmetric about the isolated-dot reference energy. Indeed, in the ordered limit, the band spans $\left[\epsilon_{\mathrm{IB}}^{\left(0\right)}-4t_{0},\;\epsilon_{\mathrm{IB}}^{\left(0\right)}+12t_{0}\right]$. The observed disorder-induced asymmetry may therefore reflect the combined effect of this intrinsically asymmetric lattice dispersion, the nonlinear radius dependence of the on-site energies, and the exponential distance dependence of the hopping amplitudes. The present calculations do not separate these contributions quantitatively, so no unique microscopic mechanism is assigned.

### 4.4. Physical Disorder Scales

Radius fluctuations generate an on-site-energy standard deviation $\sigma_{E}$, while both radius and positional fluctuations contribute to the hopping dispersion. To compare the on-site-energy disorder with a characteristic kinetic energy scale, we introduce a moment-based effective kinetic bandwidth $W_{\mathrm{eff}}$.

Following the spectral-moment approach of Gaspard and Cyrot-Lackmann [79], we consider the kinetic Hamiltonian $H_{\mathrm{kin}}$, constructed from the same hopping network as the full Hamiltonian but with vanishing on-site energies. Its second spectral moment is

(54) $$\mu_{2}=\frac{1}{N}\mathrm{Tr}\;\left(H_{\mathrm{kin}}^{2}\right)=\frac{2}{N}\sum_{(i,j)\in E}t_{ij}^{\;2}.$$

We then define the characteristic kinetic bandwidth scale as

(55) $$W_{\mathrm{eff}}=4\sqrt{\mu_{2}}.$$

**Figure 5:**
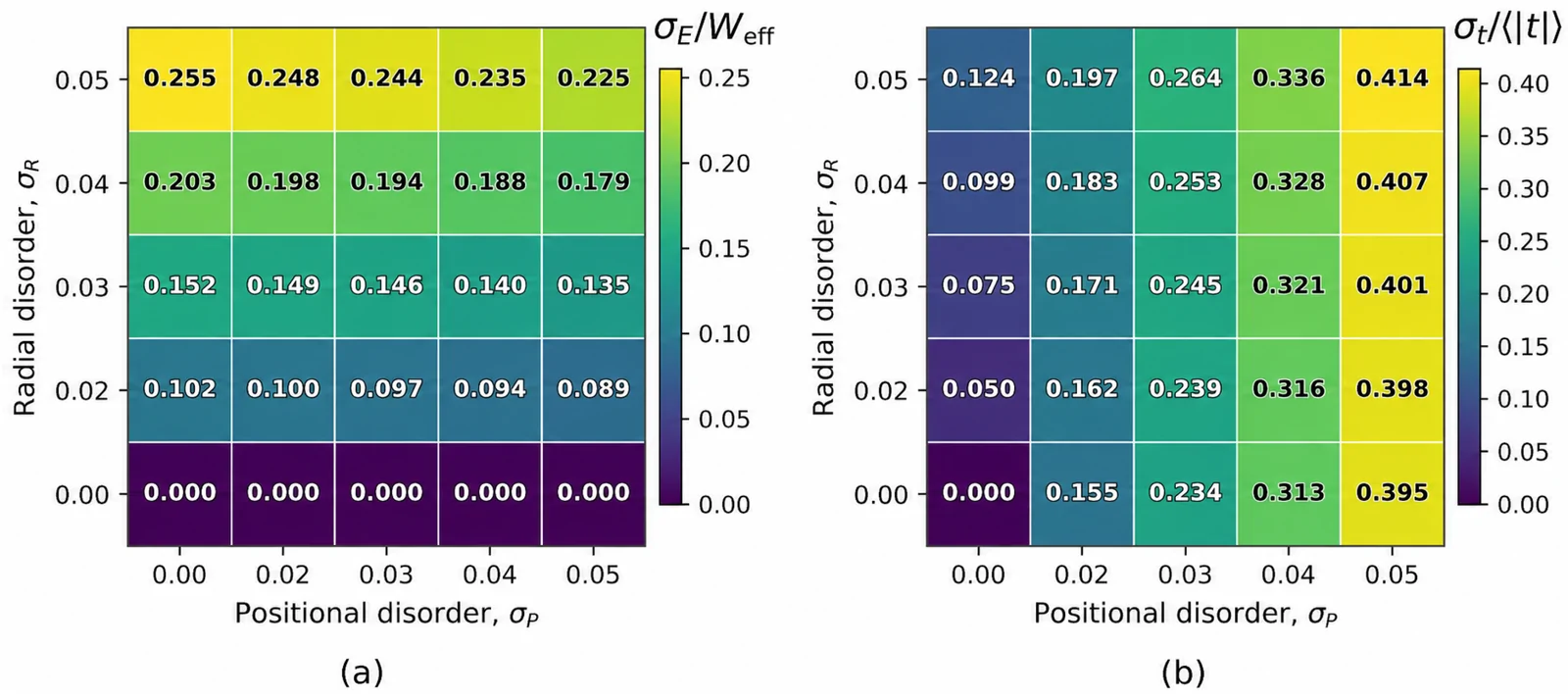
Ensemble-averaged disorder scales: (**a**) normalized on-site-energy dispersion $\sigma_{E}/W_{\mathrm{eff}}$; and (**b**) relative hopping-magnitude dispersion $\sigma_{t}/\langle |t|\rangle$.

This quantity is a second-moment estimate of the characteristic kinetic bandwidth and should not be interpreted as the exact spectral span of the disordered Hamiltonian. The normalized disorder scales shown in Figure 5 are therefore

(56) $$\frac{\sigma_{E}}{W_{\mathrm{eff}}},\;\frac{\sigma_{t}}{\langle |t|\rangle }.$$

Figure 5 displays these two normalized disorder scales.

The scale $\sigma_{E}/W_{\mathrm{eff}}$ varies predominantly with $\sigma_{R}$ and is zero when all radii are identical. For $\sigma_{P}=0$, its approximate values are 0.102, 0.152, 0.203, and 0.255 for $\sigma_{R}=0.02$, 0.03, 0.04, and 0.05, respectively. Small changes with $\sigma_{P}$ at fixed $\sigma_{R}$ arise because the denominator $W_{\mathrm{eff}}$ is realization dependent. They should not be interpreted as changes in the absolute on-site-energy variance without examining $\sigma_{E}$ separately.

The relative hopping dispersion varies mainly with $\sigma_{P}$, consistent with the exponential dependence of tunnelling on interdot separation. For example,

(57) $$\frac{\sigma_{t}}{\langle |t|\rangle }≃0.395$$

for $\left(\sigma_{R},\sigma_{P}\right)=\left(0,0.05\right)$, compared with approximately 0.124 for (0.05,0) and 0.414 for (0.05,0.05). Radius disorder nevertheless contributes to hopping disorder through both the surface separation and the radius-dependent decay length.

### 4.5. Finite-Size Analysis

The mean effective participation number of the threshold-defined core is

(58) $$N_{\mathrm{eff},\mathrm{core}}^{\left(0.5\right)}=N\Pi_{\mathrm{core}}^{\left(0.5\right)}.$$

We fit the finite-size data to

(59) $$N_{\mathrm{eff},\mathrm{core}}^{\left(0.5\right)}=CN^{\alpha },$$

where α=1 would correspond to extensive scaling and α=0 to a size-independent effective participation number.

**Figure 6:**
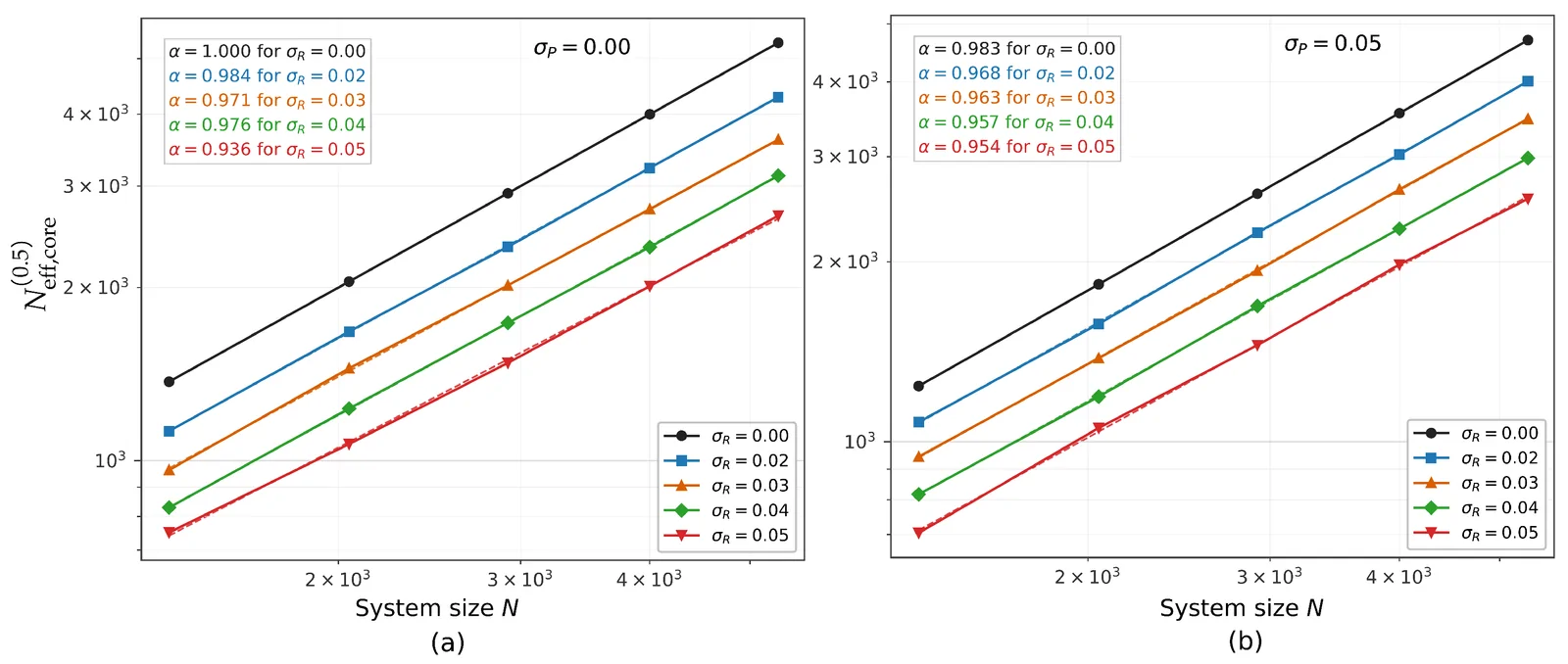
Finite-size scaling of $N_{\mathrm{eff},\mathrm{core}}^{\left(0.5\right)}=N\Pi_{\mathrm{core}}^{\left(0.5\right)}$ versus *N* for nominal radius-disorder amplitudes $\sigma_{R}=0.00$, 0.02, 0.03, 0.04, and 0.05: (**a**) $\sigma_{P}=0$; and (**b**) $\sigma_{P}=0.05$. Dashed lines are power-law fits, and the annotations report the fitted exponents α.

Figure 6 shows the log–log data and power-law fits. For $\sigma_{P}=0$, the fitted exponents are approximately 1.000, 0.984, 0.971, 0.976, and 0.936 as $\sigma_{R}$ increases from 0 to 0.05. For $\sigma_{P}=0.05$, the corresponding values are approximately 0.983, 0.968, 0.963, 0.957, and 0.954. Over the investigated finite-size range, these values are consistent with near-extensive growth of the effective core, with a decreasing participation prefactor as disorder increases. Because several exponents are below unity and the available size range is limited, these fits do not establish a non-zero normalized participation in the thermodynamic limit.

Since

(60) $$\Pi_{\mathrm{core}}^{\left(0.5\right)}=CN^{\alpha -1},$$

a nearly size-independent curve is consistent with α≃1.

**Figure 7:**
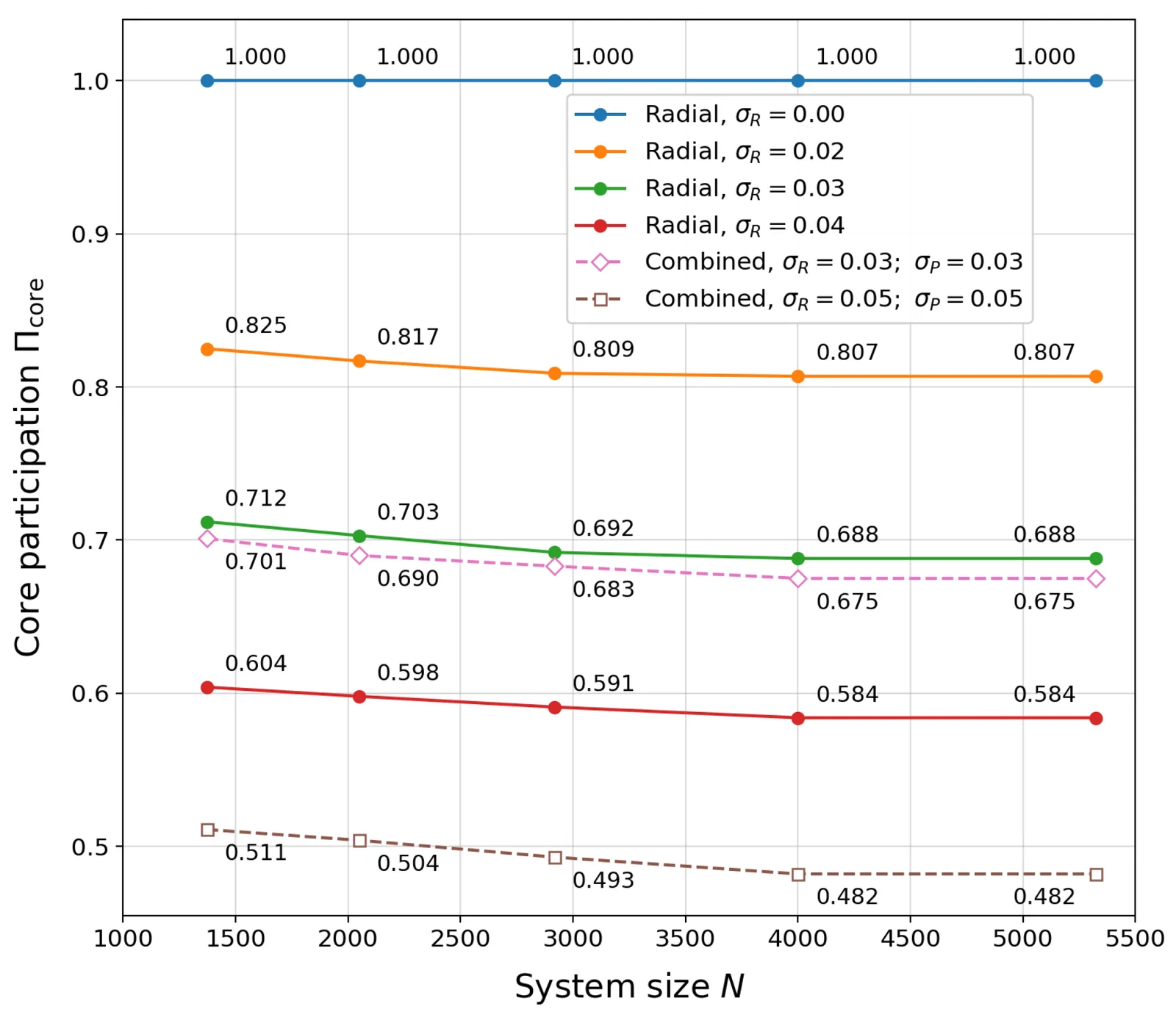
Normalized core participation as a function of system size for representative disorder cases. The weak size dependence over the simulated range is a finite-size diagnostic consistent with near-extensive scaling. Note that it should not be interpreted as proof of a non-zero thermodynamic-limit plateau.

Figure 7 shows only moderate decreases over the simulated sizes. The apparent flattening at the two largest sizes is compatible with the fitted near-unity exponents, but two closely spaced large-*N* points are insufficient to demonstrate an asymptotic plateau.

The finite-size calculations were performed for 15 independent disorder realizations for each $\left(N,\sigma_{R},\sigma_{P}\right)$ point. Since the N=4000 values shown in Figure 7 are obtained from an independent set of 15 disorder realizations, they may therefore differ by a few units in the third decimal from the corresponding entries of Figure 2, which are averaged over 20 realizations. This occurs in the combined cases, which differ by approximately 0.005 from those in Figure 2. Thus, the values reported in Figure 7 at N=4000 are consistent with the corresponding entries of the disorder map in Figure 2 within the realization-to-realization scatter of the two independent ensembles.

The present finite-size calculations show near-extensive scaling over the simulated system sizes, but they do not establish a non-zero normalized participation in the thermodynamic limit, locate a mobility edge, or exclude a slower crossover at larger *N*. A definitive thermodynamic-limit study would require numerical techniques that avoid full dense diagonalization and access substantially larger length scales.

One route could be the transfer-matrix method applied to quasi-one-dimensional bars. For a transverse size *M*, the energy- and disorder-dependent quasi-one-dimensional localization length $\lambda_{M}$ can be computed and the reduced quantity $\lambda_{M}/M$ analyzed by one-parameter finite-size scaling. Size-independent crossings provide a standard route to mobility-edge estimation and to the critical localization-length exponent ν [80]. A complementary strategy is to use sparse shift-invert Lanczos or Arnoldi eigensolvers to target narrow energy windows in much larger three-dimensional samples. The resulting wavefunctions can then be analyzed by multifractal finite-size scaling of generalized participation ratios, allowing simultaneous estimation of critical and multifractal exponents [81]. Energy-level statistics, including the crossover between Wigner–Dyson-like and Poisson-like behavior, would provide an additional independent localization diagnostic. These methods would be required to establish a thermodynamic mobility edge. Please note that the present participation fits are intentionally restricted to finite-size trends.

## 5. Discussion

### 5.1. Interpretation of the Model Results

The computed disorder maps support an energy-selective degradation picture for the adopted model. As radius disorder increases, participation is reduced first near the spectral edges, whereas a more strongly participating central region persists over a wider disorder range. Radius disorder therefore produces two simultaneous effects: it lowers the participation of the most extended states and contracts the energy interval over which comparatively extended states are retained. Consequently, the total IB width is not, by itself, a sufficient indicator of the spatial quality of the intermediate band.

The three proposed descriptors provide complementary information about this degradation. The core participation $\Pi_{\mathrm{core}}^{\left(0.5\right)}$ measures the mean spatial participation of the states belonging to the selected contiguous core, whereas $\eta_{0.5}$ measures the fraction of the total IB spectral width occupied by the selected contiguous core. Their product, $A_{\mathrm{IB}}^{\left(0.5\right)}$, decreases when either the spatial participation or the retained energy support is reduced. It should nevertheless be regarded as a compact descriptor of the one-particle eigenstate structure rather than as a transport coefficient or a photovoltaic performance metric.

For the baseline parameter set, radius disorder produces a markedly larger response than positional disorder at equal nominal parameter values. Positional disorder alone leaves the combined descriptor relatively high, with $A_{\mathrm{IB}}^{\left(0.5\right)}≃0.857$ at $\left(\sigma_{R},\sigma_{P}\right)=\left(0,0.05\right)$. By comparison, radius disorder alone gives $A_{\mathrm{IB}}^{\left(0.5\right)}≃0.222$ at (0.05,0), while the combined case (0.05,0.05) gives approximately 0.144.

This comparison must be interpreted within the definitions of the disorder variables and the adopted effective parameters. Equal numerical values of $\sigma_{R}$ and $\sigma_{P}$ do not correspond to equal realized variances, equal geometrical perturbations, or equal microscopic energy scales. The results therefore establish a ranking within the sampled disorder map, rather than a universal comparison between radius and positional disorder in arbitrary quantum-dot ensembles.

The results obtained are consistent with the structure of the Hamiltonian. Radius fluctuations modify the confinement-dependent on-site energies and also affect the tunnelling amplitudes through changes in surface separation and wavefunction decay. Positional fluctuations act mainly through the distance dependence of the tunnelling terms. However, because radius disorder changes diagonal and off-diagonal Hamiltonian elements simultaneously, the present simulations do not independently determine which of these two channels is responsible for the observed degradation. Separating their contributions would require controlled calculations in which on-site-energy disorder and hopping disorder are varied independently.

The energy-resolved participation curves further show that the degradation is not a uniform downward displacement of the participation throughout the spectrum. At moderate radius disorder, states near the central part of the intermediate band remain substantially more extended than states near its edges. At stronger radius disorder, both the maximum participation and the extent of this central region are reduced. The numerical curves also exhibit an asymmetry around the IB center. The present calculations establish this asymmetry as a feature of the model response but do not identify a unique microscopic origin for it.

Within the selected baseline parameterization, these results indicate that quantum-dot size uniformity is a more restrictive model control variable than moderate positional fluctuations for preserving a spatially extended IB over the simulated system sizes. This conclusion concerns one-particle eigenstate participation. It does not directly predict optical absorption, carrier mobility, conductivity, recombination rates, current–voltage characteristics, or photovoltaic conversion efficiency.

### 5.2. Scope and Limitations

The model is an effective one-particle representation intended to isolate the influence of structural disorder on IB eigenstates. Each quantum dot is represented by a single effective orbital, while the perovskite matrix enters only through effective on-site and tunnelling parameters. Spin degeneracy, Coulomb charging, many-body correlations, excitonic effects, electron–phonon coupling, surface-trap manifolds, explicit host valence and conduction bands, and spatial variations in matrix composition or barrier height are not included. The calculated descriptors therefore characterize the spatial structure of the model eigenstates rather than the complete optical, transport, recombination, or photovoltaic response of a device.

The parameters $A_{R}$, $t_{0}$, $\lambda_{0}$, $f_{\mathrm{CQD}}$, $s_{\min}$, and the neighbor cutoff define a particular effective reference system. The apparent crossover around $\sigma_{R}=0.03$–0.04 should consequently be interpreted as a feature of this parameterization. It is not a universal manufacturing tolerance. Its position and sharpness may change with confinement strength, tunnelling barrier, quantum-dot volume fraction, connectivity, surface chemistry, or the assumed dependence of the effective parameters on quantum-dot radius.

The threshold q=0.5 is an operational convention analogous to a half-maximum construction. It provides a reproducible definition of a contiguous core centered on the maximum-participation state but does not define a critical localization boundary. Since the threshold is defined relative to $\Pi_{\max}$, the selected core depends on the energy-resolved shape of the participation profile. A systematic study of alternative *q* values would be required to quantify the sensitivity of the numerical crossover to this convention.

The energy-resolved curves in Figure 4 are expressed in a zero-centered energy coordinate obtained by referencing each realization to its own spectral midpoint before ensemble averaging. It does not enter the calculation of the eigenstates or participation descriptors.

The robustness of the observed asymmetry has been tested using a rank-based measure independent of the energy origin, while a complementary mirror comparison quantifies the asymmetry of the ensemble-averaged profile relative to $E_{\mathrm{ref}}$.

The descriptor $\eta_{0.5}$ is an energy-width fraction and should not be interpreted as the fraction of eigenstates contained in the core. Likewise, $A_{\mathrm{IB}}^{\left(0.5\right)}$ is a composite descriptor that combines both spatial participation and spectral support. It is not an observable efficiency, conductivity, mobility, or absorption coefficient.

The finite-size calculations show that the effective number of quantum dots participating in the selected core grows approximately extensively over the simulated size range, with fitted exponents close to unity. As discussed in Section 4.5, these fits do not demonstrate a non-zero normalized participation in the thermodynamic limit, locate a mobility edge, or exclude a slow crossover at larger sizes. Larger systems, additional disorder realizations, and explicit uncertainty estimates for the fitted exponents would be needed for a more stringent finite-size analysis.

A further limitation is the neglect of the charging energy and many-body correlations. Transport studies of lead-chalcogenide CQD arrays show that weakly coupled systems can exhibit Coulomb blockade and hopping conduction, and that increasing interdot coupling changes the transport regime [82]. More generally, the electronic state of a CQD solid is controlled by the competition among charging energy, energetic disorder, and interdot coupling [83]. If the screened on-site interaction *U* becomes comparable to or larger than the relevant collective kinetic bandwidth, particularly near a commensurate filling, Coulomb blockade or Mott-like localization may occur even when the corresponding non-interacting eigenstates are spatially extended. The present analysis should therefore be regarded as a necessary single-particle structural condition for preserving a collective IB manifold, not as a sufficient condition for metallic or band-like transport. A correlated extension would require an Anderson–Hubbard [73] or extended-Hubbard description with screened on-site and inter-site interactions and an explicit carrier filling.

The disorder treated in this work is quenched structural disorder. No dynamical lattice degrees of freedom are included, so the calculations represent a frozen-geometry baseline rather than the finite-temperature transport of an operating device. Electron–phonon coupling can dynamically modulate both on-site energies and tunnelling amplitudes, introduce dephasing and reorganization energies, and lead to polaron formation. First-principles calculations for PbS-nanocrystal solids with X-type surface ligands have reported reorganization energies of tens to hundreds of meV and electronic couplings smaller than the reorganization energy, resulting in localized polarons and phonon-assisted charge transfer [76]. Those values are not directly transferable to the present PbS/perovskite effective model, but they demonstrate that lattice reorganization can be a competing energy scale in lead-chalcogenide CQD systems.

Dynamic lattice effects could therefore reduce the coherence length, shift the apparent structural-disorder crossover, or mask it in a finite-temperature transport measurement. Conversely, phonons may also assist transitions between localized states, so their effect cannot be represented simply as an additional static localization rate. A finite-temperature extension could combine thermally sampled configurations or molecular-dynamics snapshots with explicit electron–phonon coupling, for example, through a Holstein- or Fröhlich-type Hamiltonian [73], followed by Kubo, master-equation, or non-equilibrium transport calculations.

The proposed descriptors can nevertheless be related qualitatively to experimentally accessible observables. Radius-induced energetic disorder and contraction of the participating spectral core can contribute to broadening and reshaping sub-bandgap optical features, consistent with the established sensitivity of QD optical spectra to size dispersion [62]. However, a state with low spatial participation can still possess finite optical oscillator strength. Absorption can therefore remain appreciable in a spectral region whose states contribute poorly to long-range carrier extraction. A useful experimental signature would consequently be a growing mismatch between sub-bandgap absorption and sub-bandgap External Quantum Efficiency/Incident Photon-to-Current Efficiency or photocurrent as disorder increases: absorption would probe the presence of optically accessible in-gap states, whereas photocurrent additionally requires sufficiently connected states and favourable extraction kinetics.

Similarly, reduced participation is qualitatively consistent with weaker electronic connectivity and a stronger tendency toward hopping-dominated transport, but it is not itself a carrier mobility. A quantitative mobility calculation requires carrier occupations, scattering and phonon dynamics, and an explicit transport formalism [73] such as Kubo, Landauer, Non-Equilibrium Green’s Function [84,85], or kinetic hopping. Temperature-dependent conductivity or mobility and time-resolved photoconductivity would therefore provide complementary experimental tests of the transport regime suggested by the participation analysis. The computed quantities should thus be regarded as structural electronic indicators to be compared with, rather than substituted for, optical and transport observables.

Future work should consequently explore the sensitivity to the effective model parameters and to the core threshold, separate diagonal and off-diagonal disorder through controlled ablations, extend the finite-size calculations, and connect the participation descriptors to optical transitions, carrier transport, recombination, and device-level photovoltaic performance.

## 6. Conclusions

We have investigated the effects of structural disorder on a quantum-dot-derived intermediate band using a single-orbital tight-binding model on a dilated face-centered-cubic lattice. Radius dispersion and positional fluctuations have been introduced separately and jointly. Their effects have been characterized using the normalized participation ratio, the mean participation of a contiguous threshold-defined spectral core, the relative energy width of that core, and the combined descriptor $A_{\mathrm{IB}}^{\left(0.5\right)}$.

For the adopted baseline parameters, the degradation is energy selective. States near the spectral edges lose participation before the most strongly participating states in the central IB region. Increasing radius disorder reduces both the maximum participation and the energy interval over which comparatively extended states persist. The total IB width is therefore insufficient as a standalone measure of the spatial quality of the band.

At equal nominal disorder amplitudes, radius disorder produces a substantially stronger response than positional disorder within the sampled parameter map. The model gives

$$A_{\mathrm{IB}}^{\left(0.5\right)}≃0.857$$

for $\left(\sigma_{R},\sigma_{P}\right)=\left(0,0.05\right)$,

$$A_{\mathrm{IB}}^{\left(0.5\right)}≃0.222$$

for (0.05,0), and

$$A_{\mathrm{IB}}^{\left(0.5\right)}≃0.144$$

for (0.05,0.05). These values indicate that moderate positional disorder is comparatively less detrimental than radius disorder for the selected model parameters. They should not be interpreted as a comparison between equal realized disorder variances or as universal fabrication tolerances.

The sampled parameter map exhibits a comparatively robust regime near $\sigma_{R}=0.02$, a crossover around $\sigma_{R}=0.03$–0.04, and strong degradation at the larger radius-disorder amplitudes. These regimes describe the response of the adopted effective model and may shift when the confinement, tunnelling, volume fraction, connectivity, or disorder definitions are changed.

The fitted exponents remain numerically close to unity over the investigated finite-size range. They do not, however, establish a thermodynamic mobility edge or a non-zero asymptotic normalized participation.

Subject to these qualifications, the principal result is that quantum-dot size uniformity is a more restrictive model control variable than moderate positional disorder for preserving a spatially extended IB core over the simulated system sizes. Further work should test the sensitivity of this conclusion to the model parameters and threshold definition, isolate the independent contributions of on-site and hopping disorder, and relate the eigenstate-participation measures to experimentally accessible optical, transport, recombination, and photovoltaic quantities.

The numerical crossover identified here is therefore a model-dependent structural benchmark for the integrity of a single effective CQD-derived manifold. It should not be interpreted as a universal fabrication tolerance or as a sufficient condition for band-like transport in a multi-orbital, interacting, finite-temperature CQD device.

These findings identify quantum-dot size uniformity as a particularly restrictive structural control variable within the adopted single-particle model, while multi-orbital, interaction, and finite-temperature effects remain important directions for future extension.

## Figures and Tables

**Table 1:** Reference parameters and disorder map used in the numerical simulations.

Quantity	Symbol	Value
Mean CQD radius	$R_{0}$	2.0nm
CQD volume fraction	$f_{\mathrm{CQD}}$	0.14
FCC lattice constant	$a_{\mathrm{FCC}}$	9.86nm
FCC nearest-neighbor distance	$d_{\mathrm{nn}}^{\mathrm{FCC}}$	6.97nm
Reference surface separation	$s_{0}^{\mathrm{FCC}}$	2.97nm
Minimum allowed surface separation	$s_{\min}$	0.5nm
Reference hopping amplitude	$t_{0}$	5meV
Reference decay length	$\lambda_{0}$	1.0nm
Radius dependence of decay length	$\beta_{\lambda }$	−1
Core threshold	*q*	0.5
Effective radial sensitivity	$A_{R}$	200meV
Radial truncation parameter	$k_{R}$	2
Positional truncation parameter	$k_{P}$	2
Neighbor cutoff factor	$\gamma_{c}$	1.2
FCC supercell size	*L*	10
Number of CQDs	$N=4L^{3}$	4000
Nominal radius-disorder grid	$\sigma_{R}$	0.00,0.02,0.03,0.04,0.05
Nominal positional-disorder grid	$\sigma_{P}$	0.00,0.02,0.03,0.04,0.05
Realizations per grid point	—	20
Realizations in the 2D disorder map	$N_{\mathrm{real}}$	500
Supercell sizes, finite-size analysis	*L*	7, 8, 9, 10, 11
System sizes, finite-size analysis	$N=4L^{3}$	1372, 2048, 2916, 4000, 5324
Realizations per finite-size point	–	15

**Table 2:** Realized geometric disorder for the pure-disorder channels. The positional quantities are normalized by $d_{\mathrm{nn}}^{\mathrm{FCC}}$.

Nominal Amplitude	$\delta_{R}^{\mathrm{real}}$ (%)	$\delta_{P,1D}^{\mathrm{real}}$ (%)	$\delta_{P,\mathrm{RMS}}^{\mathrm{real}}$ (%)
0.00	0.000±0.000	0.000±0.000	0.000±0.000
0.02	1.758±0.017	1.566±0.006	2.712±0.010
0.03	2.641±0.025	2.345±0.010	4.063±0.017
0.04	3.513±0.027	3.124±0.018	5.411±0.031
0.05	4.418±0.046	3.904±0.013	6.761±0.023

## Data Availability

The original contributions presented in this study are included in the article. Further inquiries can be directed to the corresponding author.
